# Supplementary figures and images for: Exploring the Mechanism of 2,4-Dichlorophenoxyacetic Acid in Causing Neurodegenerative Diseases Based on Network Toxicology and Molecular Docking
Source: Int J Mol Sci. 2025 Dec 12;26(24):11980. doi: 10.3390/ijms262411980 (PMC12732694; doi:10.3390/ijms262411980)

**A**

**N  
O  
S  
3**

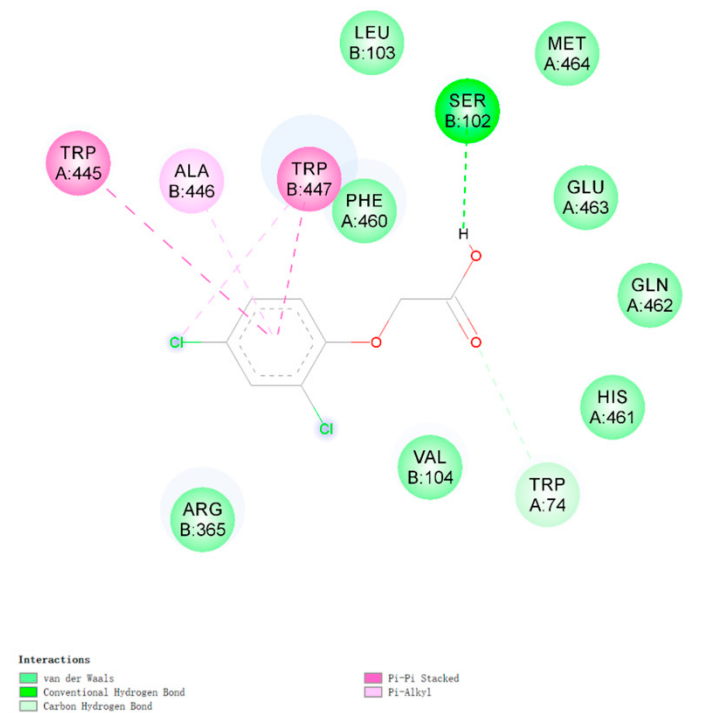

**B**

**N  
F  
K  
B  
1**

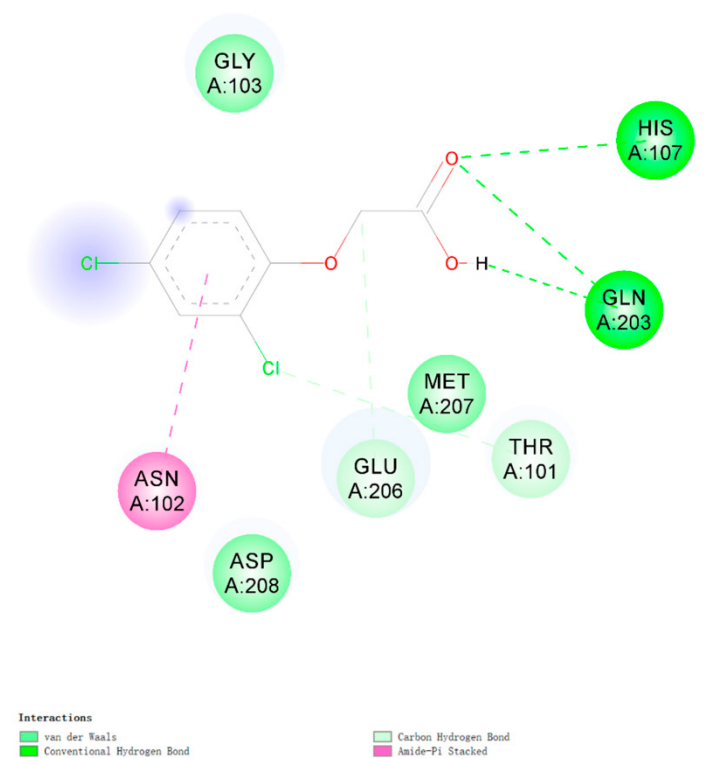

Figure S1:2D molecular docking diagram of NFKB1 and NOS3

Supplement: Supplementary file 1 [file ijms-26-11980-s001.zip › Supplementary Figure.pdf]
